# Cutaneous mast cell tumors in dogs: Association with gonadectomy

**DOI:** 10.29374/2527-2179.bjvm000726

**Published:** 2026-08-12

**Authors:** Florencia Sollier Podestá, Federico García Balbiani, Fabio Funcasta Michelena, Daniela Izquierdo Caquías, Valerie Cayssials da Cunha

**Affiliations:** 1 Unidad de Clínica y Cirugía de Pequeños Animales, Facultad de Veterinaria, Universidad de la República (UDELAR), Montevideo, Uruguay.; 2 Facultad de Veterinaria, Universidad de la República (UDELAR), Montevideo, Uruguay.; 3 Unidad de Bioestadística, Facultad de Veterinaria, Universidad de la República (UDELAR), Montevideo, Uruguay.

**Keywords:** cancer, mast cell tumor (MCT), reproductive status, canines, case-control study, câncer, tumor de mastócitos (TMC), estado reprodutivo, caninos, estudo caso-controle

## Abstract

A retrospective case-control study was conducted using the clinical records of dogs treated at the Veterinary Teaching Hospital of Uruguay between 2017 and 2022 with a confirmed diagnosis of cutaneous mast cell tumor (MCT). A total of 141 affected dogs were compared with a randomly selected control group of 141 dogs without cutaneous tumors that attended the hospital during the same period. The association between gonadectomy and cutaneous MCT was evaluated by estimating crude and sex- and age-adjusted odds ratios (ORs) using univariate and multivariable logistic regression models. Overall, gonadectomy was associated with lower odds of cutaneous MCT. However, effect modification by sex was detected in the association between gonadectomy and MCT (*p*-interaction = 0.04). Sex-stratified analyses showed that gonadectomy was significantly associated with lower odds of MCT in males (OR = 0.16, 95% confidence interval [CI], 0.06-0.41), whereas the association in females was weaker and not statistically significant (OR = 0.56, 95% CI, 0.25-1.27). In addition, dogs aged ≥7 years had substantially higher odds of MCT than adult dogs aged 2-6 years. These findings suggest that the association between reproductive status and cutaneous MCT is complex and may differ by sex. However, given the observational nature of the case-control design, the results should not be interpreted as evidence of a causal relationship. Further prospective studies, particularly those evaluating the timing of gonadectomy, are warranted.

## Resumo

Foi realizado um estudo retrospectivo de caso-controle utilizando registros clínicos de cães atendidos em um serviço de consulta veterinária entre 2017 e 2022, com diagnóstico confirmado de tumor de mastócitos cutâneo (TMC). Um total de 141 cães afetados foi comparado a um grupo controle, selecionado aleatoriamente, composto por cães sem tumores cutâneos (n = 141) atendidos no mesmo período. Para avaliar a associação entre o diagnóstico de TMC cutâneo e a gonadectomia, foram estimadas razões de chances (ORs) brutas e ajustadas por sexo e idade, utilizando regressão logística univariada e multivariada. A gonadectomia esteve associada a uma menor probabilidade de TMC. No entanto, foi identificada modificação de efeito na associação entre gonadectomia e TMC de acordo com o sexo (p de interação = 0,04). Análises estratificadas por sexo mostraram que essa associação foi significativa apenas em machos (OR = 0,16; IC 95%: 0,06-0,41), enquanto em fêmeas foi menos pronunciada e não significativa (OR = 0,56; IC 95%: 0,25-1,27). Além disso, cães com idade ≥7 anos apresentaram chances marcadamente maiores de TMC em comparação com aqueles entre 2 e 6 anos. Esses achados sugerem que a associação entre o estado reprodutivo e o mastocitoma é complexa e pode variar de acordo com o sexo. Dada a natureza observacional do estudo caso-controle, os resultados não devem ser interpretados como evidência de causalidade. Estudos prospectivos adicionais, particularmente aqueles que avaliem o momento da gonadectomia, são necessários para melhor compreender essa relação.

## Introduction

Cancer is one of the leading causes of death in both humans and dogs (Vail & MacEwen, 2000), and the skin is the organ most frequently affected by neoplasms in dogs (Dobson & Scase, 2007; Kok et al., 2019). Although most reported cutaneous tumors are benign, mast cell tumors (MCTs) are the most common malignant skin neoplasms (Martins et al., 2022). MCTs exhibit marked biological heterogeneity, ranging from solitary low-grade lesions to aggressive high-grade tumors with variable metastatic potential (Nardi et al., 2022). Histopathological evaluation and immunohistochemical analysis are the primary methods for predicting biological behavior and are essential for therapeutic decision-making and research (Willmann et al., 2021).

Several risk factors for MCT development have been investigated, including breed predisposition, body condition, sex, and reproductive status (Hart & Hart, 2021; Hart et al., 2020; White et al., 2011). In particular, increasing attention has focused on the potential influence of gonadectomy on the development of various neoplasms. Removal of the gonads disrupts the hypothalamic-pituitary-gonadal axis by eliminating gonadal hormone feedback, resulting in chronically elevated circulating luteinizing hormone (LH) concentrations (Kutzler et al., 2022). Because LH receptors are expressed not only in reproductive tissues but also in the skin (Welle et al., 2006), it has been proposed that sustained supraphysiological LH concentrations may contribute to MCT oncogenesis (Kutzler et al., 2022). However, evidence regarding the association between reproductive status and MCT risk remains inconsistent. Some studies have reported no association between reproductive status and MCT development (Mochizuki et al., 2017; Shoop et al., 2015), whereas others have found that gonadectomy in both males and females is associated with an increased risk of MCT (Hart et al., 2020; Pierini et al., 2019; White et al., 2011). Similarly, Zink et al. (2014) reported that gonadectomy is associated not only with an increased risk of MCT but also with an earlier age at diagnosis. In contrast, Torres de la Riva et al. (2013) suggested that the increased risk associated with gonadectomy is greater when the procedure is performed later in life.

Given these conflicting findings and the widespread use of elective gonadectomy as a strategy for population control and public health, clarifying the relationship between reproductive status and MCT development is of considerable clinical importance. Therefore, the objective of this study was to evaluate the association between reproductive status and cutaneous MCT presentation in dogs treated at a veterinary hospital in Uruguay. To our knowledge, this is the first reported study to investigate this association in the region.

## Materials and methods

### Study design and population

A retrospective observational case-control study was conducted. All cases consisted of dogs diagnosed with cutaneous MCTs at the Veterinary Teaching Hospital of Uruguay. Dogs identified in the hospital's electronic medical database between January 2017 and December 2022 with a diagnosis of cutaneous MCT confirmed by histopathology or cytology were included (n = 141). Controls were randomly selected from the same electronic medical database during the same study period (January 2017-December 2022). They consisted of dogs without cutaneous tumors that presented to the hospital for non-neoplastic conditions, including musculoskeletal, urinary, digestive, and respiratory disorders. Thus, both cases and controls were drawn from the same source population, clinical setting, and recruitment period.

### Data collection

Recorded variables included age (categorized as ≤1 year, 2-6 years, and ≥7 years), sex, reproductive status (intact or neutered), breed, tumor location, tumor size, tumor evolution (defined as the interval between tumor appearance and clinical diagnosis), diagnostic method, and histopathologic grade according to the classification proposed by Kiupel et al. (2011). When medical records were incomplete, owners were contacted by telephone to confirm the dog's reproductive status at the time of tumor presentation.

### Statistical analysis

Descriptive statistics were used to summarize the demographic characteristics of cases and controls and the tumor characteristics of cases. Associations among tumor-related variables were assessed using chi-square tests of independence. To evaluate the association between reproductive status and MCT, dogs neutered before tumor diagnosis were classified as exposed, whereas intact dogs and those neutered after tumor diagnosis were classified as unexposed. Cases lacking definitive information on reproductive status before diagnosis (n = 15) were excluded from this analysis.

Crude (unadjusted) odds ratios (ORs) with 95% confidence intervals (CIs) were initially calculated to evaluate the association between each explanatory variable and MCT occurrence. Adjusted ORs with 95% CIs were then estimated using a multivariable logistic regression model to assess the association between gonadectomy and MCT while controlling for sex and age category. The model included an interaction term between reproductive status and sex. The significance of each variable in the multivariable model was assessed using likelihood ratio tests (LRTs) by comparing the full model with reduced models excluding each predictor. Because evidence of interaction was identified, sex-stratified logistic regression analyses were subsequently performed.

Statistical significance was defined as *p* < 0.05. All analyses were performed using Jamovi version 2.5.

## Results

### Study population

Table 1 summarizes the demographic characteristics of the study population. Both the MCT and control groups included 141 dogs. Females predominated in the MCT group (64.5%), whereas males predominated in the control group (51.1%). Gonadectomy rates were similar between groups (56.0% in cases and 62.4% in controls). Most dogs with MCT (90.0%) were ≥7 years old. Mixed-breed dogs represented the largest proportion of the study population (41.1%), followed by Labrador Retrievers (9.6%), Boxers (7.4%), and Pitbull dogs (7.4%). Golden Retrievers were more common among cases than controls (8.5% vs. 0.7%). The remaining 24.1% of the population comprised German Shepherds, Dachshunds, French Bulldogs, Cimarrons, Jack Russell Terriers, Dogo Argentinos, Shar-Peis, Greyhounds, Pointers, Weimaraners, American Staffordshire Terriers, Beagles, German Shorthaired Pointers, English Bulldogs, Rottweilers, English Mastiffs, Cocker Spaniels, Bull Terriers, Doberman Pinschers, Yorkshire Terriers, Border Collies, Dalmatians, Fila Brasileiros, Miniature Pinschers, Shih Tzus, Australian Cattle Dogs, Bichons Frisés, and Argentine Arrieros (Uruguay, 2017).

**Table 1:** Demographic characteristics of canine patients treated at the Veterinary Hospital Center (2017-2022).

Variable	Case group n = 141	Control group n = 141	TOTAL n = 282
**Sex**			
Females	91 (64.50%)	69 (48.94%)	160 (56.74%)
Males	50 (35.50%)	72 (51.06%)	122 (43.26%)
**Reproductive state**			
Neutered Females	56 (39.72%)	50 (35.46%)	106 (37.59%)
Neutered Males	8 (5.67%)	38 (26.95%)	46 (16.31%)
Intact Females	25 (17.73%)	19 (13.47%)	44 (15.60%)
Intact Males	37 (26.24%)	34 (24.11%)	71 (25.18%)
No data available^*^	15 (10.64%)	0 (0%)	15 (5.32%)
**Age**			
≤1 year	1 (0.71%)	10 (7.09%)	11 (3.90%)
2-6 years	13 (9.22%)	49 (34.75%)	62 (21.99%)
≥ 7 years	127 (90.07%)	82 (58.16%)	209 (74.11%)

* Castrated individuals in whom their reproductive status prior to the appearance of the tumor could not be established.

### Tumor characteristics

Table 2 summarizes the clinicopathological characteristics of canine cutaneous MCTs. The limbs were the most common tumor location (42.6%), particularly the hindlimbs. No significant association was found between tumor size (≤3 cm vs. >3 cm) and tumor evolution (<1 year vs. ≥1 year) (χ^2^ = 0.807, df = 1, *p* = 0.37). Histopathological grade was determined for 76 of 141 tumors (53.9%) following surgical excision using the grading system proposed by Kiupel et al. (2011). Most tumors were classified as high-grade (55.3%). Although high-grade tumors tended to be larger than 3 cm, this association did not reach statistical significance (χ^2^ = 3.41, df = 1, *p* = 0.07) (Supplementary Table 1). Likewise, no significant association was found between tumor duration and histopathological grade (χ^2^ = 0.134, df = 1, *p* = 0.72) (Supplementary Table 1).

**Table 2:** Clinicopathological characteristics of canine cutaneous mast cell tumors included in the study

**Variable**	**n (%)**
**Anatomic site (n=141)**	
Hindlimb	40 (28.37%)
Forelimb	20 (14.18%)
Trunk	24 (17.02%)
Head and neck	19 (13.47%)
Multiple	18 (12.77%)
Others^*^	20 (14.19%)
**Histological grading (n=76)**	
Low	34 (44.70%)
High	42 (55.30%)
**Tumoral size (n=141)**	
≤3 cm	91 (64.54%)
4-8 cm	34 (24.82%)
≥9 cm	13 (9.22%)
No data available	2 (1.42%)

* Other locations include: vulva and scrotum, inguinal region, breasts, perineum.

### Association between mast cell tumors and reproductive status

After excluding cases in which the temporal relationship between gonadectomy and MCT diagnosis could not be established (n = 15), the analysis included 126 cases and 141 controls. Among females, most dogs were neutered in both the MCT and control groups. In contrast, neutering was less common among males with MCT than among control males (Table 1).

In the univariable analysis, neutered dogs had lower odds of MCT than intact dogs (OR = 0.62; 95% CI, 0.38-1.01), although this association became stronger after adjustment for sex and age (adjusted OR = 0.16; 95% CI, 0.06-0.41) (Table 3).

**Table 3:** Crude (unadjusted) and adjusted odds ratio (OR, and 95% confidence interval) according to the logistic regression analysis for the associations between MCT cases and reproductive status, age and sex variables, overall and stratified by sex.

**Variable**		**Crude OR (95% CI)**	**Adjusted OR (95% CI)**	**LRT *p*-value**
***Overall***				
Reproductive status				<0.001
	Intact	1 (ref.)	1 (ref.)	
	Neutered	0.62 (0.38 - 1.01)	**0.16 (0.06 - 0.41)**	
**Age**				<0.001
	2-6 years	1 (ref.)	1 (ref.)	
	≤1 year	0.34 (0.04 - 3.22)	0.30 (0.03 - 2.64)	
	≥ 7 years	**5.15 (2.62 - 10.11)**	**5.84 (2.87 - 11.89)**	
**Sex**				0.36
	Male	1 (ref.)	1 (ref.)	
	Female	**1.88 (1.15 - 3.07)**	1.49 (0.64 - 3.47)	
**Interaction**				0.04
	Reproductive status×Sex		**3.56 (1.05 - 12.08)**	
***Male***				
**Reproductive status**				<0.001
	Intact	1 (ref.)	1 (ref.)	
	Neutered	**0.19 (0.08 - 0.47)**	**0.16 (0.06 - 0.41)**	
**Age**				<0.001
	2-6 years	1 (ref.)	1 (ref.)	
	≤1 year	---	---	
	≥ 7 years	**4.18 (1.56 - 11.20)**	**5.10 (1.79 - 14.57)**	
***Female***				
**Reproductive status**				0.16
	Intact	1 (ref.)	1 (ref.)	
	Neutered	0.85 (0.42 - 1.73)	0.56 (0.25 - 1.27)	
**Age**				<0.001
	2-6 years	1 (ref.)	1 (ref.)	
	≤1 year	0.45 (0.05 - 4.31)	0.42 (0.04 - 4.11)	
	≥ 7 years	**5.74 (2.25 - 14.59)**	**6.51 (2.48 - 17.13)**	

MCT cases (n = 126) and non-MCT controls (n = 141). The significance of each variable in the multivariate model was evaluated by a likelihood ratio test (LRT).

Dogs ≥7 years old had significantly higher odds of MCT (adjusted OR = 5.84; 95% CI, 2.87-11.89), whereas no significant association was observed for dogs ≤1 year old. Sex was not independently associated with MCT occurrence in the multivariable model (adjusted OR = 1.49; 95% CI, 0.64-3.47). However, a significant interaction was identified between sex and reproductive status (LRT for interaction, *p* = 0.04). In sex-stratified analyses, neutering was associated with significantly lower odds of MCT in males (adjusted OR = 0.16; 95% CI, 0.06-0.41), whereas no significant association was observed in females (adjusted OR = 0.56; 95% CI, 0.25-1.27).

Overall, these findings indicate that the association between gonadectomy and MCT varies by sex, underscoring the importance of accounting for effect modification by sex in the analysis (Table 3).

## Discussion

To our knowledge, this is the first reported regional study to examine the association between reproductive status and canine cutaneous MCTs, providing novel epidemiological data from a South American population. Although the case-control design is susceptible to several potential biases, including information, temporal, and survival biases, among others, and the findings should not be interpreted as evidence of causality, several of our results are consistent with previous reports. The reported mean age at onset of canine neoplasia ranges from 8 to 11 years (Elgue et al., 2012), whereas that for MCTs ranges from 7 to 10 years (Denis et al., 2020). Consistent with these findings, dogs older than 7 years had substantially higher odds of MCT than younger adults, supporting age as an important risk factor (Martins et al., 2022; Shoop et al., 2015).

Mixed-breed dogs accounted for 41.1% of the study population. Among purebred dogs, Boxers, Pit Bulls Terriers, Labrador Retrievers, and Golden Retrievers were the most frequently represented breeds. This distribution is consistent with previous studies (Denis et al., 2020; Mochizuki et al., 2017; Pierini et al., 2019; Shoop et al., 2015). Clinically, MCTs may present as solitary or multiple masses. Previous studies have reported that approximately 50% of MCTs occur on the trunk, perineal, and inguinal regions, 40% on the limbs, and 10% on the head and neck (Martins et al., 2022; Nardi et al., 2022; Welle, 2008). In the present study, only 29.8% of tumors were located on the trunk or in the perineal/inguinal regions, which may be explained by the inclusion of a separate category for multiple presentations (12.8%). The limbs were the most common anatomical location (42.6%), consistent with previous reports (Martins et al., 2022; Santos et al., 2020). Proposed explanations for the anatomical distribution of MCTs include environmental and metabolic factors (Ciążyńska et al., 2021).

Several studies have reported that tumors measuring ≤3 cm are associated with a more favorable prognosis (Nardi et al., 2022; Pierini et al., 2019). Although survival outcomes were not evaluated in the present study, smaller tumors were more frequently classified as low-grade, whereas larger tumors tended to be high-grade. However, this association did not reach statistical significance, possibly due to missing data. Likewise, no significant association was observed between tumor duration and histopathological grade, despite previous reports indicating that longer tumor duration is associated with increased malignancy (Renzi et al., 2010; Tejera-Vaquerizo et al., 2020).

Most tumors evaluated histopathologically were classified as high-grade according to the Kiupel et al. (2011) grading system. Although most published studies report a predominance of low-grade MCTs (Kiupel et al., 2011; Martins et al., 2022; Stefanello et al., 2015), our findings are consistent with those of a previous local study (Denis et al., 2020). The discrepancy with international reports may reflect differences in population characteristics or potential selection bias associated with a referral hospital population.

Most studies have found no association between sex and the risk of developing MCT (Martins et al., 2022; Shoop et al., 2015; Śmiech et al., 2018). In our study, females were overrepresented among cases, resulting in a significant association between female sex and MCT in the univariate (crude) model (OR = 1.88, 95% CI = 1.15-3.07). However, this association was no longer statistically significant after adjustment for reproductive status and age (adjusted OR = 1.49, 95% CI = 0.64-3.47), suggesting that the crude association was attributable to confounding by these covariates rather than an independent effect of sex. This result agrees with previous findings (Denis et al., 2020). One possible explanation is survival bias. A separate study conducted at the same hospital found that males with MCT had up to a 2.3-fold higher risk of death than females (Menéndez, 2024), potentially leading to an underrepresentation of male cases in this hospital-based population.

Overall, 59.2% of dogs in the study population were neutered. This prevalence is substantially higher than the 34% reported in a previous local study, highlighting variation across populations. Similarly, studies from other countries have reported considerable variability in neutering prevalence (García et al., 2018; Gomes et al., 2003; Rocha et al., 2022). Collectively, these findings suggest that neutering prevalence is influenced by population characteristics and management practices.

When evaluating the association between MCT and gonadectomy after adjusting for sex and age, neutered dogs had lower odds of MCT than intact dogs. However, a significant interaction between sex and gonadectomy indicated that the association differed by sex. Specifically, the apparent protective association of gonadectomy was attenuated in females, resulting in a non-significant association, whereas gonadectomy was associated with lower odds of MCT in males. These findings differ from those of previous studies. Zink et al. (2014) reported an increased risk of MCT in neutered dogs, whereas White et al. (2011) found higher odds in spayed females than in intact females and suggested a possible increase in risk among castrated males compared with intact males. In contrast, our results indicate lower odds of MCT associated with gonadectomy in males, whereas no significant association was observed in females. The markedly low odds of MCT observed in neutered males should be interpreted cautiously. This finding may partly reflect the distribution of the study population, as neutered males were substantially more common among controls than among cases. Furthermore, the case group was considerably older than the control group, and age is likely associated with reproductive status in the hospital population. These imbalances, together with the relatively small number of neutered male cases included in the stratified analysis, may have inflated the estimated effect size because of confounding, residual confounding, model instability, and selection bias inherent to retrospective hospital-based case-control studies.

Overall, these findings underscore the ongoing controversy regarding the role of gonadectomy in MCT development. An additional factor that may explain discrepancies between our results and those reported previously is that gonadectomy was evaluated without considering the timing of the procedure relative to sexual maturity (e.g., before versus after the first estrus in females). Previous studies have suggested that the age at gonadectomy may influence cancer risk. For example, Torres de la Riva et al. (2013) reported an increased risk of tumor development when gonadectomy was performed later in life, whereas Hart et al. (2014, 2020) and Zink et al. (2014) described heterogeneous associations that appear to depend on sex, breed, and age at surgery. Moreover, the timing of gonadectomy may differ between males and females in clinical practice, which could contribute to the sex-specific associations observed in the present study. Consequently, the absence of stratification by age at gonadectomy should be considered a limitation, particularly given the heterogeneous and sometimes contradictory evidence reported in the literature (Grüntzig et al., 2015; Pierini et al., 2019; White et al., 2011). In addition, the retrospective case-control design precludes causal inference and warrants cautious interpretation of these associations. Several biological mechanisms have been proposed to explain potential associations between gonadectomy and cancer risk. One hypothesis is that gonadectomy disrupts negative feedback within the hypothalamic-pituitary-gonadal axis, resulting in increased circulating LH concentrations. Binding of LH to its receptor, which has been implicated in cellular proliferation, has been proposed as a potential mechanism contributing to tumor development (Kutzler et al., 2022). In addition, Hoffman et al. (2013, 2018) reported an interaction between sex and gonadectomy with respect to longevity, with spayed females and intact males exhibiting longer lifespans. Because increasing age is associated with a greater likelihood of developing MCT, prolonged longevity may partly explain the higher occurrence reported in spayed females (McMillan et al., 2024). However, in the present study, age, sex, and reproductive status were simultaneously included in the multivariable model. Therefore, the higher odds of MCT observed in females relative to males were estimated after controlling for differences in age and reproductive status. Finally, Torres de la Riva et al. (2013) proposed that late gonadectomy may increase MCT risk through the loss of the modulatory effects of estrogen on previously sensitized cells. Nevertheless, these mechanisms remain speculative and cannot be evaluated within the constraints of the present study design.

## Conclusions

This retrospective case-control study identified an association between reproductive status and the occurrence of cutaneous MCT in dogs. Neutering was associated with lower odds of cutaneous MCT in males, whereas this effect was not clearly established in females. Given the inherent susceptibility of the retrospective case-control design to bias, these findings should be interpreted with caution and should not be considered evidence of a causal relationship. Further research is warranted to clarify the roles of sex and neutering in the development of cutaneous MCT in dogs.

## Supplementary Material

Supplementary material accompanies this paper.

Supplementary Table 1 Distribution of tumor grade according to time of evolution and tumor size

This material is available as part of the online article from https://doi.org/10.29374/2527-2179.bjvm000726
