# Supplementary material for: Cutaneous mast cell tumors in dogs: Association with gonadectomy
Source: Braz J Vet Med. 2026 Aug 12;48:e000726. doi: 10.29374/2527-2179.bjvm000726 (PMC13466041; doi:10.29374/2527-2179.bjvm000726)
Supplement: Supplementary file 1 [file bjvm-48-e000726-suppl.pdf]

Supplementary Table 1. Distribution of tumor grade according to time of evolution and tumor size

| Variable / Category | High grade (%) | Low grade (%) |
|---|---|---|
| **Time of evolution** | | |
| **< 3 months** | 7.89% | 14.47% |
| **3-5 months** | 15.79% | 5.26% |
| **6 months - 1 year** | 19.74% | 14.47% |
| **> 1 year** | 7.89% | 7.89% |
| **No data available** | 3.95% | 2.63% |
| **Tumor size** | | |
| **≤ 3 cm** | 30.00% | 34.00% |
| **4-8 cm** | 18.00% | 6.00% |
| **≥ 9 cm** | 5.00% | 4.00% |
| **No data available** | 1.00% | — |

**Note:** Percentages are calculated based on the total number of cases included in the analysis.
